# On the Febrifuge Properties of Salicine

**Published:** 1855-12

**Authors:** 


On the Febrifuge Properties of Salicine. By Dr. Macari.—Salicine is a white, crystalline, soluble substance, and is exclusively employed in Spain and Portugal in the treatment of intermittents. As there is reason to believe its importance as an indigenuous febrifuge has been underrated, or that its employment has not been properly managed, it may be interesting to quote the conclusions Dr. Macari, a Sardinian practitioner, has arrived at respecting it.

1. Salicine is an important succedaneum to quinine, both from its febrifuge power, and the repugnance many patients manifest to quinine.
2. The dose is from 15 to 45 grains, given during the intervals of a paroxysm, and repeated according to circumstances.
3. The first dose generally notably diminishes the intensity and duration of the next paroxysm, but rarely cuts the fever short.
4. The mode of action is analogous to that of quinine, but feebler, so that the drug is not suited to pernicious fever liable to fatal termination.
5. It is given most efficaciously in solution.
6. Its price, already much lower than that of quinine, is susceptible, in the event of a demand arising, of much diminution, owing to the little value of willow bark.
7. Salicine does not give rise to any of the perturbations of the nervous system producible by the preparations of bark.
8. It is successful in all simple intermittent fevers, at whatever age, or state of health, these may occur; and it may, therefore, be often used as a substitute for quinine, when this becomes distasteful or inefficient. There are cases, however, which resist its influence, and for which quinine is required.—(*Jour. de Pharm.*) *London Lancet.*

